# Supplementary material for: Exploring the influence of competition on arbovirus invasion risk in communities
Source: PLoS One. 2022 Oct 12;17(10):e0275687. doi: 10.1371/journal.pone.0275687 (PMC9555654; doi:10.1371/journal.pone.0275687)
Supplement: S1 File — (PDF) [file pone.0275687.s001.pdf]

S1 File: Including latency periods in vector species

The system (1) in the main text can be extended to include the latency period in the vectors, that is, the time for viral incubation. Vectors, when infected, go to the latent compartment and if they do not die of natural causes can then transit into the infectious compartment. Given that vectors have a relatively short lifespan, latency periods can represent a substantial fraction of their lives, thus influencing transmission. For simplicity, we ignore vertical transmission in vectors in this section.

The equations for the total abundances N_i remain the same, as well as the equations for the infected hosts I_3 and I_4. The rest of the system then becomes

$$
\begin{aligned}
E_1' &= \left(\beta_{13}\frac{\lambda_1 I_3}{\lambda_1 N_3 + N_4} + \beta_{14}\frac{I_4}{\lambda_1 N_3 + N_4}\right)S_1 \\
&\quad - (c_{11}d_{11}N_1 + c_{12}d_{12}N_2 + \mu_1 + \gamma_1)E_1 \\
E_2' &= \left(\beta_{23}\frac{\lambda_2 I_3}{\lambda_2 N_3 + N_4} + \beta_{24}\frac{I_4}{\lambda_2 N_3 + N_4}\right)S_2 \\
&\quad - (c_{21}d_{21}N_1 + c_{22}d_{22}N_2 + \mu_2 + \gamma_2)E_2 \\
I_1' &= \gamma_1 E_1 - (c_{11}d_{11}N_1 + c_{12}d_{12}N_2 + \mu_1)I_1 \\
I_2' &= \gamma_2 E_2 - (c_{21}d_{21}N_1 + c_{22}d_{22}N_2 + \mu_2)I_2
\end{aligned}
$$

where γ_i corresponds to the transition rate (per day) from the latent, E_i, to infectious, I_i, compartments for vector species i. The model (1) presented in the main text is a special case of the one above, when $\gamma_i \to \infty$.

Following the same methodological steps as before for the full system, we arrive at the Next Generation Matrix of large domain $\boldsymbol{K_L}$ [1,2]

$$
\boldsymbol{K_L} = \begin{pmatrix}
0 & 0 & 0 & 0 & k_{15} & k_{16} \\
0 & 0 & 0 & 0 & k_{25} & k_{26} \\
0 & 0 & 0 & 0 & 0 & 0 \\
0 & 0 & 0 & 0 & 0 & 0 \\
k_{51} & k_{52} & k_{53} & k_{54} & k_{55} & 0 \\
k_{61} & k_{62} & k_{63} & k_{64} & 0 & k_{66}
\end{pmatrix}
$$

with elements

$$k_{15} = \frac{\beta_{13}\lambda_1 N_1}{(\lambda_1 N_3 + N_4)(\alpha_3 + \mu_3 + c_{33}d_{33}N_3 + c_{34}d_{34}N_4)}$$
$$k_{16} = \frac{\beta_{14}N_1}{(\lambda_1 N_3 + N_4)(\alpha_4 + \mu_4 + c_{43}d_{43}N_3 + c_{44}d_{44}N_4)}$$
$$k_{51} = \frac{\beta_{31}\lambda_1 N_3\gamma_1}{(\mu_1 + c_{11}d_{11}N_1 + c_{12}d_{12}N_2)(\lambda_1 N_3 + N_4)(\mu_1 + c_{11}d_{11}N_1 + c_{12}d_{12}N_2 + \gamma_1)}$$
$$k_{52} = \frac{\beta_{32}\lambda_2 N_3\gamma_2}{(\mu_2 + c_{21}d_{21}N_1 + c_{22}d_{22}N_2)(\lambda_2 N_3 + N_4)(\mu_2 + c_{21}d_{21}N_1 + c_{22}d_{22}N_2 + \gamma_2)}$$
$$k_{53} = \frac{\beta_{31}\lambda_1 N_3}{(\mu_1 + c_1 1 d_1 1 N_1 + c_1 2 d_1 2 N_2)(\lambda_1 N_3 + N_4)}$$
$$k_{54} = \frac{\beta_{32}\lambda_2 N_3}{(\mu_2 + c_2 1 d_2 1 N_1 + c_2 2 d_2 2 N_2)(\lambda_2 N_3 + N_4)}$$
$$k_{55} = \frac{\beta_{33}N_3}{(N3 + N_4)(\mu_3 + c_{33}d_{33}N_3 + c_{34}d_{34}N_4 + \alpha_3)}$$
$$k_{61} = \frac{\beta_{41}N_4\gamma_1}{(\mu_1 + c_{11}d_{11}N_1 + c_{12}d_{12}N_2)(\lambda_1 N_3 + N_4)(\mu_1 + c_{11}d_{11}N_1 + c_{12}d_{12}N_2 + \gamma_1)}$$
$$k_{62} = \frac{\beta_{42}N_4\gamma_2}{(\mu_2 + c_{21}d_{21}N_1 + c_{22}d_{22}N_2)(\lambda_2 N_3 + N_4)(\mu_2 + c_{21}d_{21}N_1 + c_{22}d_{22}N_2 + \gamma_2)}$$
$$k_{63} = \frac{\beta_{41}N_4}{(\mu_1 + c_{11}d_{11}N_1 + c_{12}d_{12}N_2)(\lambda_1 N_3 + N_4)}$$
$$k_{64} = \frac{\beta_{42}N_4}{(\mu_2 + c_{21}d_{21}N_1 + c_{22}d_{22}N_2)(\lambda_2 N_3 + N_4)}$$
$$k_{66} = \frac{\beta_{44}N_4}{(N_3 + N_4)(\mu_4 + c_{43}d_{43}N_3 + c_{44}d_{44}N_4 + \alpha_4)}$$

This can be reduced to the Next Generation Matrix by focusing on the states-at-infection only, rather than on all infected states [2]. This matrix is then given by

$$\boldsymbol{K_L} = \begin{pmatrix} 0 & 0 & k_{13} & k_{14} \\ 0 & 0 & k_{23} & k_{24} \\ k_{31} & k_{32} & k_{33} & 0 \\ k_{41} & k_{42} & 0 & k_{44} \end{pmatrix}$$

Again, the dominant eigenvalue of $\boldsymbol{K}$ corresponds to R_0 and gives the epidemiological stability of the disease-free steady state [2].

Fig SS1 shows the effect of competition between the different mosquito species and between the bird species on R_0, accounting for the latency period in the mosquitoes. The patterns are the same as those observed in Fig 3 in the main text, except with lower values for R_0.

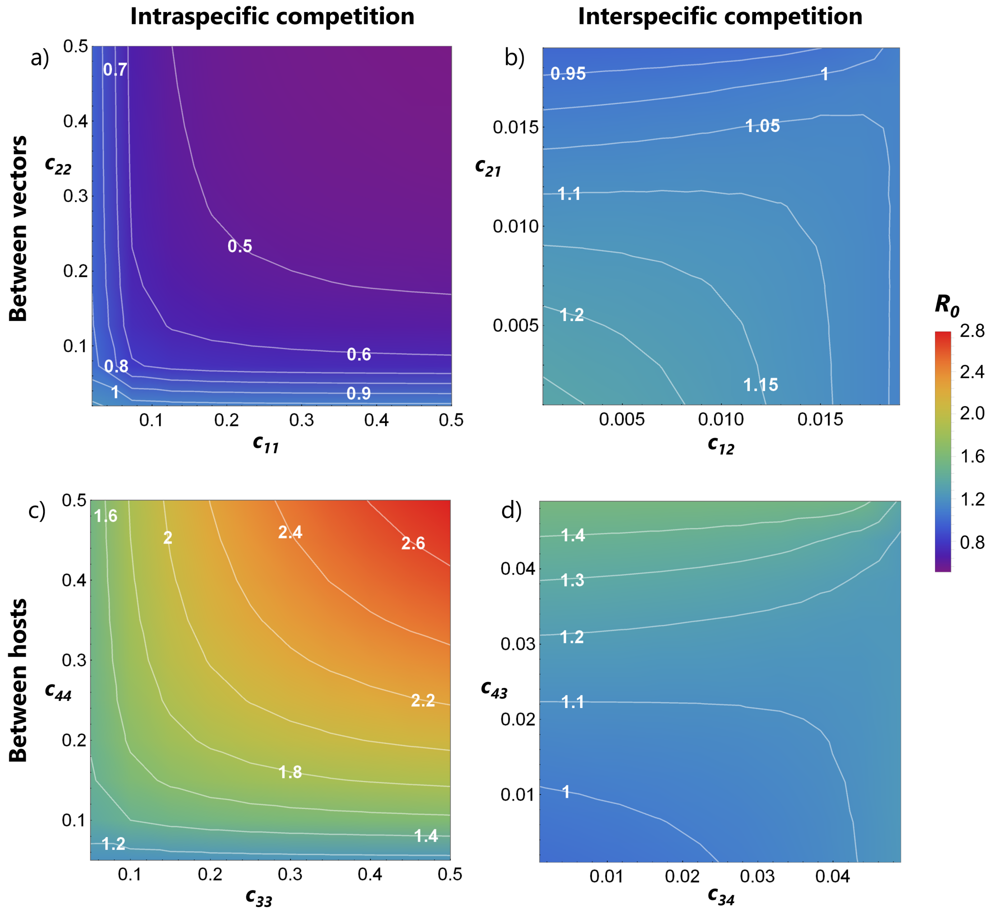

Fig S1. R_0 **values based on intraspecific (a and c) and interspecific competition (b and d) between vectors (a and b) and between hosts (c and d), assuming latency periods in both vector species.** All other parameters are fixed to the values in Table 1 and with $q_1 = q_2 = 0$, and $\gamma_1 = \gamma_2 = 0.107$ [3].

References

1. Diekmann O, Heesterbeek JAP. Mathematical epidemiology of infectious diseases: model building, analysis and interpretation. vol. 5. John Wiley & Sons; 2000.

2. Diekmann O, Heesterbeek JaP, Roberts MG. The construction of next-generation matrices for compartmental epidemic models. Journal of The Royal Society Interface. 2010;7(47):873–885. doi:10.1098/rsif.2009.0386.

3. Hartemink Na, Davis Sa, Reiter P, Hubálek Z, Heesterbeek Jap. Importance of Bird-to-Bird Transmission for the Establishment of West Nile Virus. Vector-Borne and Zoonotic Diseases. 2007;7(4):575–584. doi:10.1089/vbz.2006.0613.
