# Supplementary material for: Exploring the influence of competition on arbovirus invasion risk in communities
Source: PLoS One. 2022 Oct 12;17(10):e0275687. doi: 10.1371/journal.pone.0275687 (PMC9555654; doi:10.1371/journal.pone.0275687)
Supplement: S2 File — (PDF) [file pone.0275687.s002.pdf]

S2 File: Relationship between competition, the overall vector-to-host ratio, and the basic reproduction number

To understand better how competition affects the overall v/h-ratio, we show in Fig S2 the result of several combinations of the competition coefficients. The ranges for intraspecific competition in vectors and in hosts are shorter since this can not exceed the interspecific competition values. Again, all remaining parameters were fixed at values collected from the literature on WNV, found in Table 1 in the main text. Stronger competition in vectors results in lower v/h-ratios, as expected since the number of vectors is decreasing. The opposite is seen when hosts compete the most. In both vectors and hosts, the relationship between competition and the resulting v/h-ratios are more non-linear for intra than for interspecific competition. The intraspecific competition between hosts is responsible for the widest change in v/h, and consequently on the largest variation of R_0 values from our model.

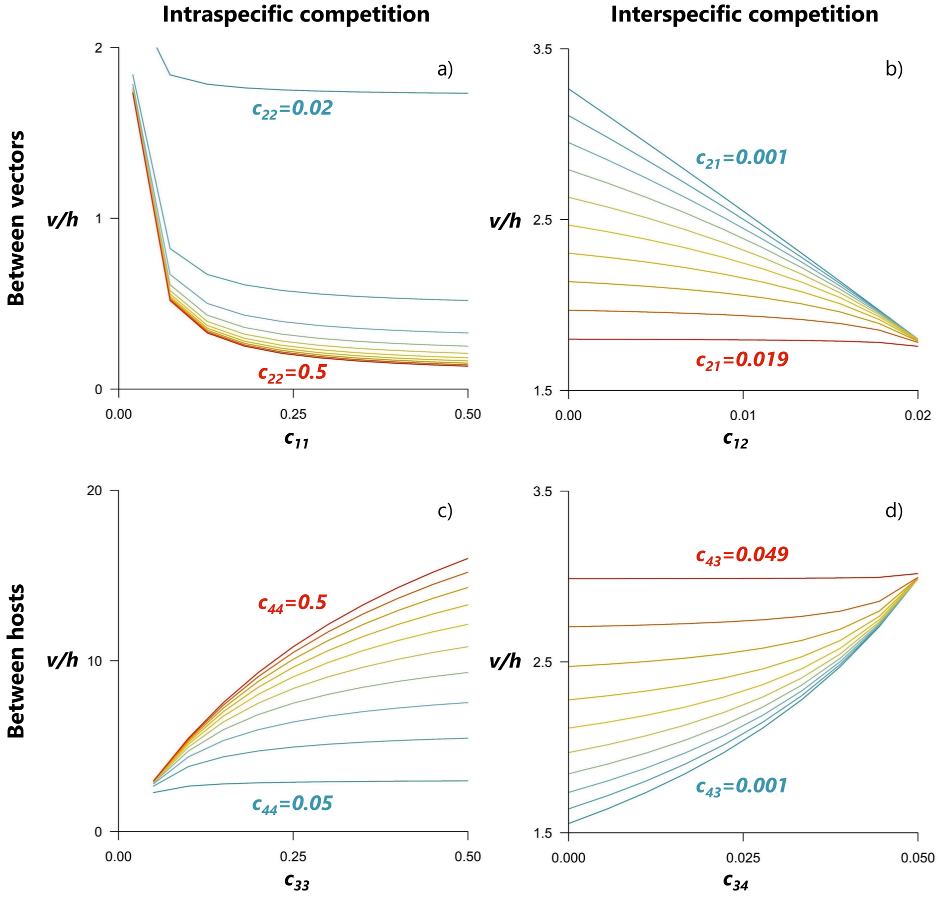

Fig S2. Non-linear effects of intraspecific (a, c) and interspecific competition (b, d) on the overall vector-to-host ratio $v/h = (N_1 + N_2)/(N_3 + N_4)$. The strength of competition ranges from relatively weak (blue lines) to relatively strong (red lines). Note that the range for the axis are different for each subplot.
