# Supplementary material for: Exploring the influence of competition on arbovirus invasion risk in communities
Source: PLoS One. 2022 Oct 12;17(10):e0275687. doi: 10.1371/journal.pone.0275687 (PMC9555654; doi:10.1371/journal.pone.0275687)
Supplement: S3 File — (PDF) [file pone.0275687.s003.pdf]

S3 File: Elasticity of the basic reproduction number to model parameters

To further measure how R_0 responds to parameter changes, we carried out an elasticity analysis following the approach of Caswell [1] and later applied to epidemiological systems by Matser et al. [2]. The sensitivity of R_0 to a given parameter informs us how a change in that parameter will affect the value of R_0. The elasticity is a similar concept, but with the added advantage of being a relative measure. This makes it a more useful metric when comparing parameters measured in very different scales.

The elasticity of R_0 to a vital rate k_{ij} (i.e., k_{ij} is a cell of the $\boldsymbol{K}$ matrix) and elasticity of R_0 to an individual lower-level parameter a, respectively, are given by

$$e_{ij} = \frac{k_{ij}}{R_0}\frac{\partial R_0}{\partial k_{ij}} \quad \text{and} \quad e(a) = \frac{a}{R_0}\sum_{ij}\frac{\partial R_0}{\partial k_{ij}}\frac{\partial k_{ij}}{\partial a}. \tag{1}$$

The elasticities to the individual parameters are calculated numerically in R using the `popbio` package using the values used in the main text. Fig S3 shows the elasticity values of R_0 to each parameter.

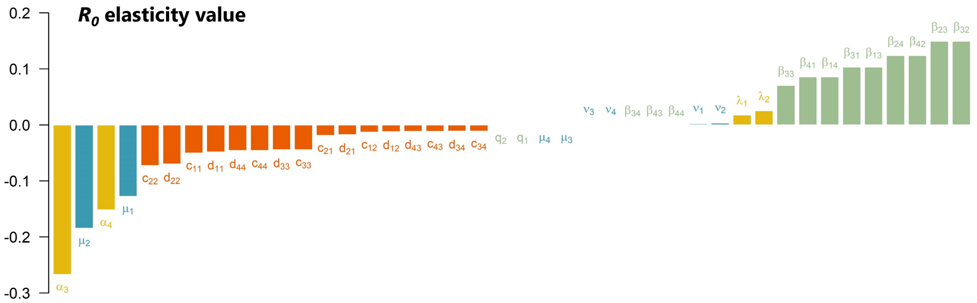

Fig S3. Elasticity values of R_0 to the individual model parameters, $e(a)$. Blue: demography parameters, red: competition parameters, green: transmission parameters, yellow: others.

R_0 is the most elastic to the additional death rates caused by WNV in the hosts, their natural death rates, and the transmission rates. Like in [2, 3] we interpret the values of the elasticities in the following way: a positive elasticity of R_0 to a particular parameter means that an increase in the value of that parameter gives an increase in R_0, while a negative elasticity gives a decrease in R_0, assuming the values of all other parameters are unchanged.

Changes in the interspecific competition between hosts leads to the largest decrease in R_0. This is consistent with the results in the main text. It should be noted that changing the competition coefficients also changes the species abundances N_i, which the elasticity analysis keeps fixed by default. Therefore the predicted R_0 changes may be underestimated using this method. Additionally, the vertical transmission in mosquitoes contributes little to changing the invasion risk (low R_0 elasticities to q_1 and q_2). Two reasons for this are the low estimates for vertical transmission in *Culex pipiens* and the fact that mosquitoes suffer a birth rate reduction due to intra and interspecific competition (at proportion $1 - d_{ii}$ and $1 - d_{ij}$, respectively). These mechanisms could also, however, have a stronger impact at different stages in time, for example, the vector vertical transmission could play an insignificant effect during summer but be relevant during overwintering.

References

1. Caswell H. Matrix Population Models: Construction, Analysis, and Interpretation. 2nd edn Sinauer Associates. Inc, Sunderland, MA. 2001;.

2. Matser A, Hartemink N, Heesterbeek H, Galvani A, Davis S. Elasticity analysis in epidemiology: an application to tick-borne infections. Ecology Letters. 2009;12(12):1298–1305. doi:10.1111/j.1461-0248.2009.01378.x.

3. Chitnis N, Hyman JM, Manore CA. Modelling vertical transmission in vector-borne diseases with applications to Rift Valley fever. Journal of Biological Dynamics. 2013;7(1):11–40. doi:10.1080/17513758.2012.733427.
