# Supplementary material for: Exploring the influence of competition on arbovirus invasion risk in communities
Source: PLoS One. 2022 Oct 12;17(10):e0275687. doi: 10.1371/journal.pone.0275687 (PMC9555654; doi:10.1371/journal.pone.0275687)
Supplement: Attachment — Submitted filename: Response to Reviewers.pdf [file pone.0275687.s004.pdf]

Response to Reviewers

Afonso Dimas Martins, Quirine ten Bosch, Hans Heesterbeek

We thank the reviewers for the thorough feedback. In our responses we refer to lines in the manuscript *with tracked changes*, to facilitate finding the adjustments.
The manuscript does not contain any data – all figures are the result of numerical simulations, with all parameter values described in the main text and tables. To further facilitate figure reproduction, we added additional details on the ranges used to generate our figures.

Reviewer 1:

Overall, this manuscript tackles an interesting problem and attempts to incorporate biologically important complexity into a disease model for WNV. I appreciate that in this work the authors chose to include important complexity into their model (multi-host/multi-vector species with intra- and interspecific competition and vector feeding preferences) to better understand WNV dynamics, and that at times other assumptions and simplifications needed to be made. However, my main suggestion is that these assumptions be better justified and their implications discussed. Along these lines, I also would have appreciated more information/clarification on WNV-specific biology which would add value to this manuscript for a wider readership.

Major comments:

Lines 18-28: Well-written justification for the study.

Lines 75-77: Assumption that birth of vectors is independent of host species abundance. This is a reasonable assumption when looking at short time scales of transmission, which could be applicable in this system under certain circumstances. However, because this assumption could heavily influence the results, I would like more discussion of why it is a valid choice and some addition to the discussion section of other potential outcomes if this assumption were not made.

Our assumption to ignore density dependence of the vector birth rate on host abundance is justified by the short time scale and the purpose of our analysis, where we only regard the invasion phase and the effects on that of competition and including non-competent/non-host species present in the ecosystem. Our aim is to show that ecological interactions may influence conclusions. We do that in the commonly adopted approaches for these systems and in situations where the vector and the pathogen have no additional impediments to their establishment. Surely, when host abundances are sufficiently low, the birth of vectors could be affected and with that the vector abundances, vector-to-host ratios and the R_0. This could lead to situations where the pathogen cannot invade even in the absence of competition, outside the interest of our current study. We assume therefore that the host abundance is sufficiently high to not limit vector feeding. There is no technical necessity to assume that density dependence in birth rate is absent. We have added the following clarification: "Effectively we assume that the hosts available for vector feeding are not limiting in abundance. That is, they are present in such numbers that all vectors can become satiated." (lines 86-89).

Lines 86-88: Again, these could be reasonable assumptions given the questions you are asking, but some justification of these choices (even with just citations) would be helpful.

There is evidence indeed that the extrinsic incubation period (i.e., latency period in vectors)

and the intrinsic incubation period (in hosts) may have an impact in WNV transmission risk (for example Foppa, I. M., & Spielman, A. 2007). Both were left out of the model because we reasoned that they would not influence the qualitative patterns of R_0 as a function of competition and we wanted to study situations where the pathogen could in principle invade. Reviewer 2 also explicitly asks for the effect of including a latency period in the vector species. Triggered by the fact that both reviewers have commented on this, we decided to test our intuition that a latency state would not qualitatively influence the patterns we observe, although such a state would lower the overall values of R_0. As the influence of a vector latency is probably the strongest, given the comparable order of the life span and the latency period, we expanded our model with a latency state in both vector species. The extended model and the companion figure to Figure 3 in the main text have now been added to the Supporting Information (Figure S1). Indeed, the resulting patterns are qualitatively similar, at lower overall values of R_0.

Lines 89-95: Choosing host-frequency dependent transmission for the model is a crucial assumption that greatly impacts the results regarding the influence of host competition on R0. Frequency-dependence is a valid choice for this system, but choosing differently could have resulted in very different final results. I suggest adding a citation on these lines to support frequency dependence, and then elaborating in the discussion section the strengths and limitations of this approach compared to others (see Wonham et al Ecology Letters 2006 for in depth examination).
This is certainly a valid point. We agree that frequency-dependence is an appropriate choice for this system but that this choice, as any choice, also has limitations. The manuscript surely benefits from referring to these differences and we have added a brief discussion of these choices (lines 592-598).

Table 1: This is the only place you mention the actual vector/host species you are using for your model (C. pipiens/molestus and the American crow/House finch). Given the complexities of the WNV system, I'd suggest a brief discussion of the biology of WNV in these chosen species somewhere in the methods and why these were chosen in particular.
In the paragraph beginning in line 278 we also mention the species we study here, but indeed we do not go in detail as to why we made these choices. In a nutshell, these species were chosen given the amount of data available on them for the WNV system. This avoids the most gaps when parameterizing the model for a common setting where WNV can invade. We mention the species we have chosen in the first paragraph of the section 'Numerical example: impact of competition on WNV invasion risk' (lines 278-281). An additional short discussion of potential impacts of focusing on different species has now been added as suggested (lines 546-563).

Lines 170-171: Here the authors make the assumption that mosquitos do not bite other species when investigating the importance of vector feeding preference. However, this negates the base model assumption made in lines 75-77 and discussed above. Given that the influence of host competition forms the crux of the interesting results from this model extension, it seems inappropriate to layer these opposing assumptions on top of one another. I would suggest either very clear justification from the authors on why these apparently diametric choices are acceptable, or re-considering the base assumption that host abundance will not impact the vector population.

As explained above (comment to lines 75-77 in the original manuscript) we indeed were not clear about this. We have clarified the issue in the text where we state the assumptions and have deleted those lines. In our basic model, we only regard the two host species, and we assume that they are not limiting vector feeding. Alternatively, if we wanted to consider additional host choices, we would indeed need to take that into account when modelling vector feeding preferences.

Figure 5: In general, I found this figure confusing and do not feel it added much value. Maybe it would be more useful as a supplementary figure for some readers. If the authors decide to keep it, please make the y axis for b and d begin at zero and add labelling for hosts vs vectors and intra vs interspecific competition like the labels in Figure 3.
Our motivation behind this figure was to have an explicit connection between the competition coefficients and the WNV invasion risk (as quantified by R_0). We considered it incomplete to not show how the competition parameters affect the vector-to-host ratio (v/h) and its respective effect on R_0. In other words, this Figure connects the results in old Figure 3 and old Figure 4. We agree, however, that this may be obvious for some readers, so we moved this Figure to the Supporting Information. On another note, the y-axis in b) and d) do not start at zero in order to easily distinguish the different lines (that would otherwise be much closer together). We added a note to the legend to remind the reader of the fact that the x and y-axis are different for each subplot. We agree that adding additional labelling as in Figure 3 helps the reader and have made that change (Fig S2).

Minor comments:
Line 3, edit to qualify the statement since isn't true of all vectors, depending on how you define a vector: "Vectors such as mosquitos usually feed on animals as they often require a blood meal for completing their lifestyle".
Agreed. Plants and fungi can also be vectors for vector-borne diseases, yet they do not behave at all like mosquitoes. To avoid confusion, we have changed this line to "Mosquito vectors feed on animals as they require a blood meal for completing their life-cycle" (lines 3-4).

Figure 3: The authors stated that they had to choose different color scales for each panel due to the differences in R0, but in looking at the range of results it seems R0 ranges from 0.6-4 across all panels. I think a color scale could be chosen such that each panel was on the same scale and therefore the results were easier to understand within and between each panel. However, if with some experimentation the authors did not think this was possible then I would suggest at least using two distinct scales, one for between vectors and one for between hosts and making those two scales distinct (white to red or white to blue, for example) for visual clarity.
We agree with the reviewer and have remade all figures using one scale for R_0 per figure to facilitate comparisons between the different panels in the same figure at a glance. To facilitate further, we have added isolines for a range of selected values of R_0. We hope that this improves comparison and interpretation of the figures.

Lines 348-349: semantics, but a relationship is either linear or not; something being "more non-linear" is kind of meaningless as a standalone statement.

Thanks for spotting that. The figure has been moved to the Supporting Information and its description was re-phrased (see section 'Relationship between competition, the overall vector-to-host ratio, and the basic reproduction number' in the Supporting Information).

Line 359: I think this is a typo and should read "In the bottom right both prefer N4".
There is indeed a typo here. The description in lines 377-378 of "in the bottom left both prefer N3" was changed to "in the bottom left both prefer N4".

Reviewer 2:
The authors present a computational exploration of the effects that intra- and interspecific competition between host and vector species have on parasite invasion into an ecosystem (community) (R0). They developed a mathematical model that includes up to two hosts and two vectors (three if you count the scenario with a dead-ends host and vector), and were able to explore how varying various ecological parameters (e.g. competition coefficients and vector to host ratios) influenced R0. I found their presentation of the mathematics required for this study are very clear, understandable, and very well explained. I appreciate the generality of their approach but also their desire to apply their model to a real system (West Nile Virus). However, I have some comments on some aspects of the model, study, and manuscript in general:

In their model, the authors assume no latency for host or vector species. While I can understand this for the host species (as relative to their lifespan their incubation period may be rather short), I believe the model should include a latent period for the vectors. As the latent period for vectors **could** represent a long proportion of the adult lifespan of the vector, all individuals that become infected may not reach the time of infectiousness (i.e. the time they are able to retransmit/pass on the parasite). However, for your model or explorations, there are multiple ways you could include this in your already developed model. For example, you could design make this binary process where either you assume the vector has a latent period or does not. At the very least this should be discussed in the discussion section as it is a common aspect of many vector-borne parasite models. Additionally, is there an extrinsic incubation period for the WNV system in hosts and/or vectors?
We agree and have performed additional analysis. See response to the comment on lines 86-88 of the original manuscript by Reviewer 1.

The authors assume that births of vector species are independent of host species abundance (line 75-80) and vectors have other feeding opportunities to meet the requirements of their lifecycle. This is an understandable simplifying assumption. However, later in the manuscript (line 170-171) the authors state that this is a closed system and that the mosquito does not bite any other animal. This seems to be the opposite of the earlier assumption.
Good point, we have fixed this. See response to the comment on lines 75-77 and lines 170-171 of the original manuscript by Reviewer 1 for elaboration.

I'm not sure why the authors assume that the "non-competent hosts" were dead-end hosts. I believe the authors had two options when modeling non-competent hosts either: 1) dead-end host approach, hosts are able to become infected but do not pass on the parasite or completely wasted bites, hosts cannot become infected but are fed on by infected vectors essentially **completely** wasting that bite from the point of view of the parasite. Why was

one chosen over the other? It seems that choosing the dead-end host approach would allow those infected individuals to be included in the expression (matrix) for R0. Perhaps, some discussion of this decision and how the other ("completely wasted bites" scenario) may play out would be useful.
In the section 'Including non-competent vectors and hosts' we make a systematic distinction between the different types of species in the ecosystem in relation to the vector and the pathogen. Nothing in the basic framework we use (Roberts & Heesterbeek, 2013) precludes any of these types. Vector transmission adds another layer of complexity because species can be non-host to the pathogen and 'non-host' to the vector and various combinations. For an initial exploration with vector-borne pathogens we have chosen to restrict ourselves to one type: the dead-end hosts. From the point of view of the pathogen and the vector, these would be the same as species that are not susceptible but that do act a source of blood meals for the vector. There is hence no epidemiological difference between 'wasted bites' and 'completely wasted bites'. Including types that do not attract (and hence feed) the mosquito at all would be an additional step, as these species only indirectly influence pathogen dynamics through their ecological interactions. We have chosen not to include such species in our analysis here because they are likely to be competing less with the competent host species. We have added a short point to the Discussion on this (lines 527-545).

I found the discussion to be lacking in how these findings relate back to previous literature and our current understanding of how competition affects parasite transmission in vector-borne parasites. Reading through the discussion seemed to be largely a recap of the results with very little reference to the literature.
We agree that the Discussion could be improved in that respect, and we have made adjustments (see for example lines 426-428 on the impact of competition; 442-445 on the relationship between competition and the vector-to-host ratio; 494-506 on the contribution of non-competent species; 592-598 on transmission assumptions).

One interesting aspect that was not discussed much in the discussion are differences in phenology between host and vector species. The current approach assumes that there are no differences in births (or deaths) through time. However, many host-vector systems have different degrees of phenological overlap between host species, vector species, and both. In particular, this could be important for the WNV system with migratory bird species and mosquito emergence. While I understand the focal topic of this paper is the effects of competition, a discussion of host and vector phenology should be included.
Indeed, we do not include seasonality in our model. The potential for invasion, as quantified by us with R_0, depends on various ingredients and several of these have values that vary with temperature, among other factors. The invasion potential will therefore vary. R_0 is not defined when ingredients vary in time. When variation is periodic, a mathematical definition exists (see the book by Diekmann, Heesterbeek and Britton for details). Alternatively, one could calculate R_0 for each separate month of the year, fixing ingredients within each month. It is unclear what would express invasion potential best because one should then also take into account variation in introduction of the pathogen. By ignoring seasonality here, we basically assume that the patterns of change in R_0 resulting from ecological interaction will be affected less by seasonal variation than the range of values that R_0 takes. We have explained this now more clearly in the Discussion and refer to some of its consequences. The reviewer gives an

example of how vectors and hosts react differently to phenology, and this is a good suggestion that we also included in the Discussion (lines 564-583).

Minor comments

I found it interesting that the authors refer to the hosts and vectors community (which is what their model represents) as an ”ecosystem” rather than a ”community”.

The words could both be used. We have a slight preference for the word ‘ecosystem’ to make explicit that we study the set of species of interest consisting of both host and non-host species to the pathogens we consider, including ecological interactions between all species. For us, this reflects a broader meaning than the word ‘community’ that in epidemiological models usually denotes a set of host species. We have clarified this in the text (lines 13-16).

Some of the lines in the model diagram (Fig 1 and 2) are hard to see. In particular the grey, yellow and green lines.

Agreed. We have changed those colours to facilitate a quick interpretation and make them more accessible.

I appreciate the inclusion of horizontal transmission for the hosts (i.e. a direct transmission route). This increases the generality of the model that could easily be ”turned off” by setting the beta parameter to 0. However, why was the transmission only considered to be intraspecific and not between different hosts (the authors briefly mention crows as an example)? Additionally, how common is this route in WNV transmission?

We did not find evidence of direct (horizontal) transmission between different species. They may be included at will in the general model, but we focus on crows and there is only evidence of horizontal intraspecific transmission. A reference for this assumption has been added to the text (line 117). It is, as expressed by our parameterization, a relatively uncommon transmission route.
