# Supplementary material for: Exploring the influence of competition on arbovirus invasion risk in communities
Source: PLoS One. 2022 Oct 12;17(10):e0275687. doi: 10.1371/journal.pone.0275687 (PMC9555654; doi:10.1371/journal.pone.0275687)
Supplement: Attachment — Submitted filename: Response to Reviewers.pdf [file pone.0275687.s005.pdf]

Response to Reviewers

Afonso Dimas Martins, Quirine ten Bosch, Hans Heesterbeek

We thank the reviewers for their critical reading of our manuscript and for their remaining comments.

Reviewer #1: The authors addressed most critiques of the reviewers thoroughly. Some additional comments below, but if the authors address these comments, at the discretion of the editor, I believe the manuscript would be ready for publication without another round of review.

Major comments:

1. On this reading, I realized that the authors focus on the idea of invasion risk, and thus make certain model assumptions around that, but never really discuss in the introduction why they focus on this point in an outbreak. Ideally the answer won't be "because it is mathematically expedient", but something tied to the interesting biology of the WNV system, or how they imagine this multi-host/multi-vector framework would be extended to other systems, etc. This doesn't need to be a huge addition, just a sentence or two to guide the reader why this is an interesting focus when they first specify that they want to focus on invasion risk (around line 50).

We agree that a brief justification for the focus on the invasion risk would be helpful and so we added that to the Introduction (lines 53-58): "Like many other vector-borne infections, WNV is expanding its range, with small outbreaks in areas that are at the borders of the current endemic range. For such small outbreaks it is important to understand how local conditions can be conducive to onward transmission. For these reasons, we focus in this study on invasion risk, rather than on long-term dynamics."

2. In the first round of review the other reviewer made a comment that the discussion section was too focused on rehashing the findings of the manuscript and not connecting it to the broader literature or scientific implications. On this reading, I agree with that comment and I do not feel the authors have addressed it. While they have added some information and new references, it is all still in the context of their own findings. I would love to see some of the repetition from the results section cut down in the discussion section and instead more ties added to the broader field, implications of this study in the context of invasion risk in other systems, etc.

We have cut some redundancy from the Discussion. We have expanded the connections to previous studies (lines 442-444: "This is in agreement with earlier modeling work on malaria transmission, which indicated that both increased mosquito competition and an increase in dead-end hosts can contribute to lower invasion risks [37]") and added broader implications (lines 620-633: "However, this model can easily be adapted to other vector-borne diseases. Particularly, those that circulate in the same vector and host populations, such as the Usutu virus or the avian malaria parasite. More generally speaking, our understanding of invasion risks of these and other vector-borne diseases can be improved by considering ecological interactions. This work should be seen as a proof-of-concept on how we can extend a vector-borne disease model to account for more complex layers of species interactions and understanding their impacts on emergence of infections in ecosystems communities.").

Minor comments:

1. Line 15: In response to a comment from the other reviewer, the authors felt the need to justify the use of the word “ecosystem” as opposed to “community”. At the end of the day, “ecosystem” is still not a very applicable term here because the authors are not considering abiotic contributors, and I don’t think this added line makes the justification well. The authors could just use “ecological interactions” when that is what they are trying to convey.
Since this issue was raised by both reviewers, we have decided to drop the term ‘ecosystem’ and use instead ‘community’. This change also affects the title of the manuscript.

2. Line 19: “These competition forces” could be changed to “These competitive forces”
3. Line 22: “Birds compete the mostly” should likely be either “Birst compete mostly” or “Birds compete the most”
4. Line 51: Switches from present tense used to describe the manuscript in the previous two sentences to future tense “we will”. I personally prefer present tense here.
5. Line 65: “We regard” is a bit awkward; I would use the phrasing “We focus on” instead
6. Line 71-72: “A compartmental model…” passive voice. I would change to “we constructed”
7. Figure 1/2: The other reviewer commented on difficulty seeing the arrows in the figures. I still personally find the light grey hard to discern
8. Figure 6: Add labels on the figure denoting which panels are host vs. vector, similar to your other figures.
9. Lines 546-563: This was added to address my previous comment wanting more information on why these specific hosts and vectors were chosen. I like this explanation but I think it should be in the introduction or methods so that a reader is aware of it from the beginning.

We have made all these adjustments to the manuscript.
